# Molecular mechanism of the dual regulatory roles of ATP on the αγ heterodimer of human NAD-dependent isocitrate dehydrogenase

**DOI:** 10.1038/s41598-020-63425-6

**Published:** 2020-04-10

**Authors:** Pengkai Sun, Tuya Bai, Tengfei Ma, Jianping Ding

**Affiliations:** State Key Laboratory of Molecular Biology, CAS Center for Excellence in Molecular Cell Science, Shanghai Institute of Biochemistry and Cell Biology, University of Chinese Academy of Sciences, Chinese Academy of Sciences, 320 Yue-Yang Road, Shanghai, 200031 China

**Keywords:** X-ray crystallography, Enzyme mechanisms

## Abstract

Human NAD-dependent isocitrate dehydrogenase (NAD-IDH) is responsible for the catalytic conversion of isocitrate into α-ketoglutarate in the Krebs cycle. This enzyme exists as the α_2_βγ heterotetramer composed of the αβ and αγ heterodimers. Our previous biochemical data showed that the αγ heterodimer and the holoenzyme can be activated by low concentrations of ATP but inhibited by high concentrations of ATP; however, the molecular mechanism was unknown. Here, we report the crystal structures of the αγ heterodimer with ATP binding only to the allosteric site (α^Mg^γ^Mg+CIT+ATP^) and to both the allosteric site and the active site (α^Mg+ATP^γ^Mg+CIT+ATP^). Structural data show that ATP at low concentrations can mimic ADP to bind to the allosteric site, which stabilizes CIT binding and leads the enzyme to adopt an active conformation, revealing why the enzyme can be activated by low concentrations of ATP. On the other hand, at high concentrations ATP is competitive with NAD for binding to the catalytic site. In addition, our biochemical data show that high concentrations of ATP promote the formation of metal ion-ATP chelates. This reduces the concentration of free metal ion available for the catalytic reaction, and thus further inhibits the enzymatic activity. The combination of these two effects accounts for the inhibition of the enzyme at high concentrations of ATP. Taken together, our structural and biochemical data reveal the molecular mechanism for the dual regulatory roles of ATP on the αγ heterodimer of human NAD-IDH.

## Introduction

The Krebs cycle is a central part of the metabolic pathway to generate ATP through the oxidation of acetyl-CoA derived from the chemical breakdown of carbohydrates, fats and proteins. Separately, the Krebs cycle also provides metabolic intermediates for biosynthetic reactions leading to the de novo synthesis of proteins, lipids and nucleic acids. Isocitrate dehydrogenases (IDHs) are a family of enzymes responsible for the oxidative decarboxylation of isocitrate (ICT) into α-ketoglutarate (α-KG) using NAD or NADP as coenzyme with the concomitant production of NADH or NADPH. In mammals, NAD-dependent IDHs (NAD-IDHs, also called IDH3) are localized to the mitochondria and participate in the Krebs cycle. In addition, mammalian cells contain two NADP-dependent IDHs (NADP-IDHs) located to both the cytosol and the mitochondria, which have been demonstrated to play important roles in cellular defense against oxidative damage, detoxification of reactive oxygen species, and synthesis of fat and cholesterol^1–4^. The crystal structures of human cytosolic NADP-IDH (also called IDH1) and porcine mitochondrial NADP-IDH (also called IDH2) have been determined, and together with biochemical data, these enzymes have been shown to exist and function as homodimers in which both subunits are catalytic^5,6^. The activity of human IDH1 is regulated through conformational changes of the active site upon substrate binding, and the other mammalian NADP-IDHs appear to share a similar regulatory mechanism^5,7^.

Compared to the NADP-IDHs, the composition and regulation of NAD-IDHs are much more complex. Mammalian NAD-IDHs are comprised of three types of subunits in the ratio of 2α:1β:1γ with similar molecular masses of 37 kDa, 39 kDa and 39 kDa, respectively^8,9^. The α and β subunits form a heterodimer (αβ) and the α and γ subunits form another heterodimer (αγ), which are assembled into a heterotetramer (α_2_βγ) and further into a heterooctamer (the heterotetramer and heterooctamer are called the holoenzyme)^10,11^. In addition, the activities of mammalian NAD-IDHs can be regulated by many metabolic intermediates and products of the Krebs cycle via feedback activation and inhibition mechanisms. Early biochemical studies showed that the α subunit is the catalytic subunit in the holoenzyme, whereas the β and γ subunits are the regulatory subunits, and the activity of the holoenzyme is positively regulated by citrate (CIT) and ADP and negatively regulated by ATP and NADH^10,12–15^. Recently, we carried out a comprehensive biochemical study on the enzymatic properties of the α_2_βγ holoenzyme and the αβ and αγ heterodimers of human NAD-IDH^16^. We found that the αγ heterodimer exhibits similar enzymatic properties as the α_2_βγ holoenzyme and can be allosterically activated by CIT and ADP, whereas the αβ heterodimer cannot; moreover, both heterodimers can be inhibited by NADH^16^. In addition, our biochemical data showed that in the holoenzyme, both α subunits have catalytic activity; in contrast, the γ subunit plays a regulatory role, whereas the β subunit plays a structural role to facilitate the assembly of the holoenzyme^16^. Our detailed structural and biochemical studies of the αγ and αβ heterodimers further revealed the molecular mechanisms by which the αγ heterodimer can be allosterically activated by CIT and ADP and inhibited by NADH, and the αβ heterodimer cannot be regulated by CIT and ADP but can be inhibited by NADH^17–20^. Intriguingly, our biochemical data also showed that the activities of the αγ heterodimer and the holoenzyme can be activated by low concentrations of ATP, but inhibited by high concentrations of ATP^16^. However, the molecular mechanism underlying the dual regulatory roles of ATP on the αγ heterodimer and the holoenzyme remained elusive.

In this work, we determined two crystal structures of the αγ heterodimer of human NAD-IDH with the active site bound with either Mg^2+^ alone or Mg^2+^ and ATP, and the allosteric site bound with Mg^2+^, CIT and ATP (α^Mg^γ^Mg+CIT+ATP^ and α^Mg+ATP^γ^Mg+CIT+ATP^). In the α^Mg^γ^Mg+CIT+ATP^ structure, there is ATP bound to the ADP binding site of the allosteric site, which stabilizes CIT binding through Mg^2+^-mediated interactions and leads the enzyme to adopt an active conformation. This provides the structural basis for why the enzyme can be activated by low concentrations of ATP. In the α^Mg+ATP^γ^Mg+CIT+ATP^ structure, there is ATP bound to the NAD binding site of the active site, which hinders NAD binding and thus inhibits the enzymatic activity. In addition, our biochemical data show that high concentrations of ATP facilitate the formation of metal ion-ATP chelates and decrease the concentration of free metal ion available for the catalytic reaction, thus inhibiting the enzymatic activity. The combination of these two effects explains why the enzyme can be inhibited by high concentrations of ATP. Our structural and biochemical data together reveal the molecular mechanism for the dual regulatory roles of ATP on the αγ heterodimer of human NAD-IDH.

## Results

### Crystal structures of the αγ heterodimer with ATP binding only to the allosteric site and to both the allosteric site and the active site

**Figure 1:**
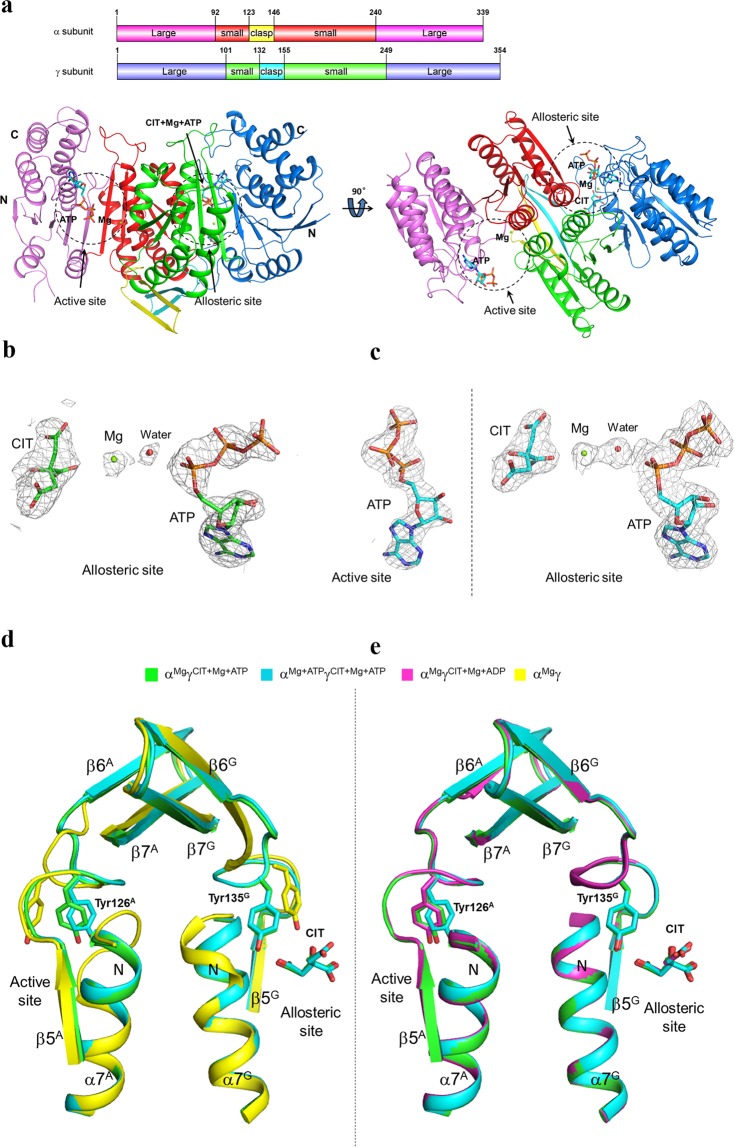
Overall structure of α^Mg+ATP^γ^Mg+CIT+ATP^ of human NAD-IDH. (**a**) Overall structure of α^Mg+ATP^γ^Mg+CIT+ATP^ in two different orientations: viewing along (right panel) and in perpendicular to (left panel) the pseudo 2-fold axis of the αγ heterodimer. The color-coding scheme of individual domains of the α and γ subunits is shown above. The bound Mg^2+^ is shown with a green sphere, and the CIT and ATP are shown with ball-and-stick models. **(b)** Representative simulated annealing composite omit map (contoured at 1.0σ level) of the bound CIT, Mg^2+^ and ATP at the allosteric site in the α^Mg^γ^Mg+CIT+ATP^ structure. **(c)** Representative simulated annealing composite omit map (contoured at 1.0σ level) of the bound ATP at the active site and the bound CIT, Mg^2+^ and ATP at the allosteric site in the α^Mg+ATP^γ^Mg+CIT+ATP^ structure. The CIT and ATP are shown with ball-and-stick models, and the Mg^2+^ and water molecules are shown with green and red spheres, respectively. **(d)** Comparison of the α^Mg^γ^Mg+CIT+ATP^ and α^Mg+ATP^γ^Mg+CIT+ATP^ structures with the inactive α^Mg^γ structure at the allosteric site, the active site, and the heterodimer interface. The color scheme of the structures is shown above. **(e)** Comparison of the α^Mg^γ^Mg+CIT+ATP^ and α^Mg+ATP^γ^Mg+CIT+ATP^ structures with the active α^Mg^γ^Mg+CIT+ADP^ structure at the allosteric site, the active site, and the heterodimer interface.

To investigate the molecular mechanism for the dual regulatory roles of ATP on the αγ heterodimer of human NAD-IDH, we initially tried to crystallize the αγ heterodimer in the presence of different concentrations of ATP, but ultimately failed to obtain any crystals of the αγ heterodimer bound with ATP alone. However, we were able to grow crystals of the αγ heterodimer with the active site bound with Mg^2+^ and the allosteric site bound with Mg^2+^, CIT and ATP from a crystallization solution containing CIT and a relatively low concentration of ATP (2 mM). This led to the determination of the structure of the α^Mg^γ^Mg+CIT+ATP^ heterodimer. Crystallization of the αγ heterodimer in solutions containing CIT and relatively high concentrations of ATP (10–20 mM) yielded crystals of the αγ heterodimer in which there is residual electron density at the NAD binding site of the active site, indicating that the NAD binding site is partially occupied by a ligand (presumably ATP, data not shown). Soaking the crystals of the α^Mg^γ^Mg+CIT^ heterodimer in a solution containing a very high concentration of ATP (200 mM) yielded crystals of the αγ heterodimer with the active site bound with Mg^2+^ and ATP and the allosteric site bound with Mg^2+^, CIT and ATP, which led to the determination of the structure of the α^Mg+ATP^γ^Mg+CIT+ATP^ heterodimer. Of note, the very high concentration of ATP (200 mM) in the soaking solution is unlikely to be physiologically relevant. Nevertheless, the structure of the α^Mg+ATP^γ^Mg+CIT+ATP^ heterodimer can provide structural insights into the mechanism by which high concentrations of ATP might compete with NAD to bind at the NAD binding site and thus inhibit the enzymatic activity of the αγ heterodimer. The α^Mg^γ^Mg+CIT+ATP^ and α^Mg+ATP^γ^Mg+CIT+ATP^ structures were determined at 2.30 Å and 2.26 Å resolution, respectively (Table 1). Both of these structures belong to space group *P*3_1_21 with each asymmetric unit containing one αγ heterodimer. In the two structures, most residues of the α and γ subunits are well defined with high-quality electron density, except for a few N-terminal and C-terminal residues and a few surface exposed loops (Fig. 1a); moreover, there are unambiguous electron densities for the bound metal ions and ligands (Fig. 1b,c). The bound metal ions were interpreted as Mg^2+^, based on the presence of Mg^2+^ in the crystallization solutions.

**Table 1:** Statistics of X-ray diffraction data and structure refinement.

Structure	α^Mg^γ^Mg+CIT+ATP^	α^Mg+ATP^γ^Mg+CIT+ATP^
PDB code	6L57	6L59
**Diffraction data**		
Wavelength (Å)	1.0000	0.9793
Space group	*P*3_1_21	*P*3_1_21
Cell parameters		
*a*, *b*, *c* (Å)	104.21, 104.21, 145.55	111.96, 111.96, 145.58
*α*, *β*, *γ* (°)	90, 90, 120	90, 90, 120
Resolution (Å)	50.0–2.30 (2.38–2.30)	50.0–2.26 (2.30–2.26)
Observed reflections	898,711	999,848
Unique reflections (I/σ(I) > 0)	41,022	50,248
Average redundancy	21.9 (20.0)	19.9 (20.7)
Average I/σ(I)	51.9 (3.4)	26.9 (3.0)
Completeness (%)	99.7 (99.3)	99.9 (99.9)
R_merge_ (%)	7.1 (79.7)	11.5 (108.0)
CC 1/2 (%)	99.5 (99.0)	99.8 (96.9)
**Refinement and structure model**		
No. of reflections (*Fo* > 0σ(*Fo*))	40,991	49,989
Working set	38,930	47,379
Test set	2,161	2,610
R_work_ / R_free_ factor (%)	19.9/23.4	20.0/22.7
Total protein atoms	4,997	4,958
Total metal atoms	2	2
Total ligand atoms	44	75
Total water atoms	125	200
Wilson B factor (Å^2^)	47.9	37.5
Average B factor (Å^2^)	56.9	50.7
Protein atoms	57.1	50.7
Metal atoms	59.4	48.8
CIT atoms	46.0	38.3
ATP atoms	63.0	65.0
Water atoms	50.2	46.4
RMS deviations		
Bond lengths (Å)	0.009	0.013
Bond angles (°)	1.0	1.0
Ramachandran plot (%)		
Most favored	97.1	96.3
Allowed	2.9	3.7
Disallowed	0	0

Our previous structural and biochemical studies demonstrated that the binding of CIT at the allosteric site induces conformational changes at the allosteric site, which are then transmitted to the active site via the heterodimer interface. This leads the active site to assume an active conformation favorable for substrate (ICT) binding, and thus decreases *S*_0.5,ICT_ and promotes the activation of the enzyme. Moreover, the binding of ADP can stabilize the CIT binding via Mg^2+^ and thus promotes the aforementioned CIT binding-induced conformational changes, which further decreases *S*_0.5,ICT_ and leads to synergistic activation of the enzyme^17^. Structural comparisons with the previously reported αγ structures show that the α^Mg^γ^Mg+CIT+ATP^ and α^Mg+ATP^γ^Mg+CIT+ATP^ structures are more similar to the active α^Mg^γ^Mg+CIT+ADP^ structure (RMSD of 0.26 Å and 0.56 Å for about 650 Cα atoms, respectively) than the inactive α^Mg^γ structure (RMSD of 1.0 Å and 1.1 Å for about 650 Cα atoms, respectively). In particular, the key residues and structure elements at the allosteric site, the active site, and the heterodimer interface all exhibit very similar conformations to those seen in the α^Mg^γ^Mg+CIT+ADP^ structure, rather than to those seen in the α^Mg^γ structure (Fig. 1d,e). For example, the side chain of Tyr135^G^ (residues of the α and γ subunits are superscripted by “A” and “G”, respectively) at the allosteric site is oriented towards the CIT-binding subsite and forms a hydrogen bond with CIT, and the side chain of Tyr126^A^ at the active site is oriented towards the ICT-binding subsite and in a proper position to interact with ICT. In both the α and γ subunits, the N-terminal region of the α7 helix at the heterodimer interface assumes an α-helical conformation to form a long α7 helix, and the β7 strand bends towards the β5-β6 loop and the α7 helix to form a network of hydrogen bonds. These results indicate that the two structures adopt an active conformation.

### ATP can bind to the ADP binding site of the allosteric site and thus activates the enzyme

**Figure 2:**
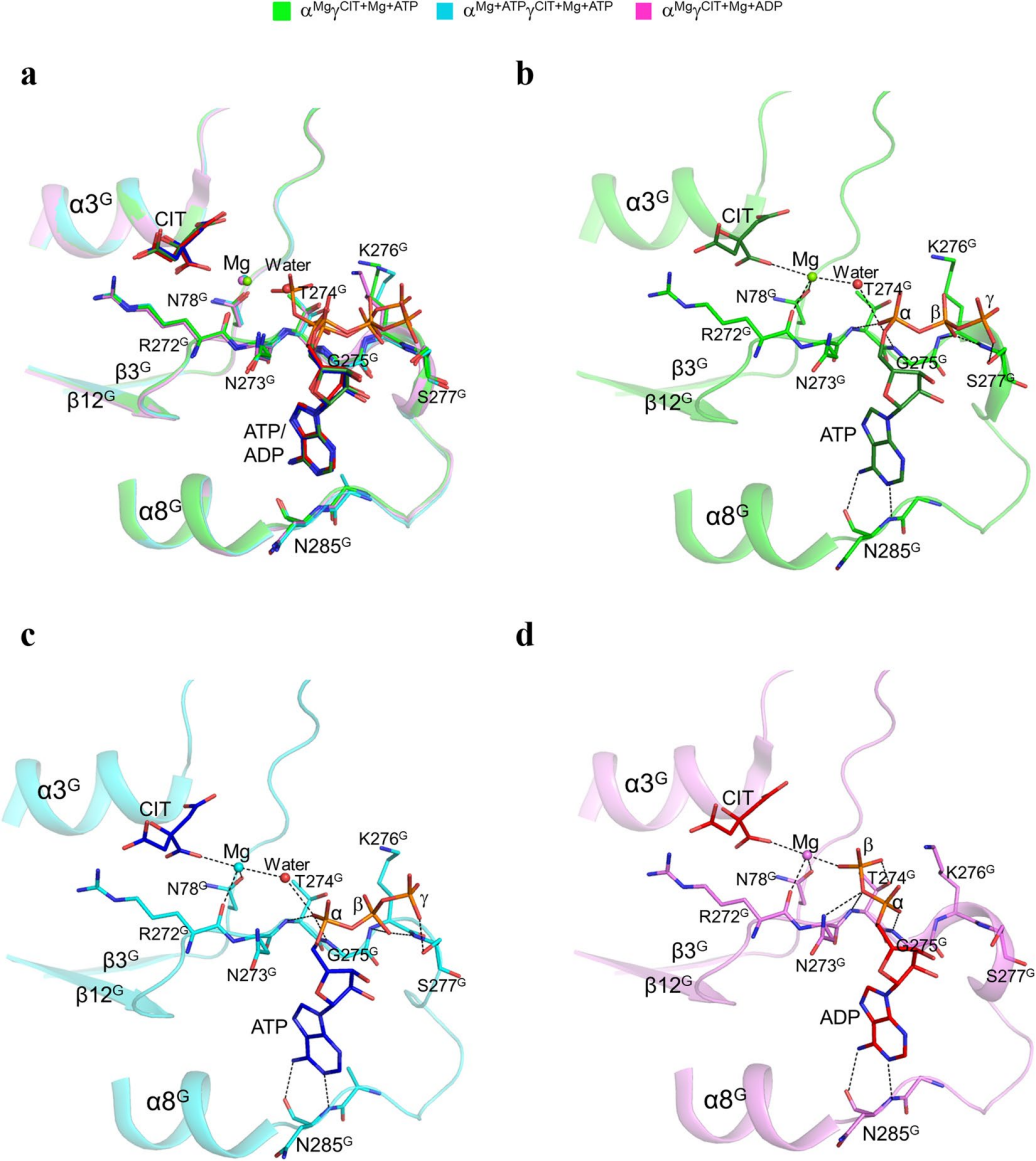
Structures of the allosteric sites in the α^Mg^γ^Mg+CIT+ATP^ and α^Mg+ATP^γ^Mg+CIT+ATP^ structures. **(a)** Comparison of the allosteric site in the α^Mg^γ^Mg+CIT+ATP^, α^Mg+ATP^γ^Mg+CIT+ATP^, and α^Mg^γ^Mg+CIT+ADP^ structures. The key residues at the allosteric site in the α^Mg^γ^Mg+CIT+ATP^ and α^Mg+ATP^γ^Mg+CIT+ATP^ structures assume very similar conformations as those in the active α^Mg^γ^Mg+CIT+ADP^ structure. The color scheme of the structures is shown above. **(b)** Structure of the allosteric site in the α^Mg^γ^Mg+CIT+ATP^ structure. **(c)** Structure of the allosteric site in the α^Mg+ATP^γ^Mg+CIT+ATP^ structure. **(d)** Structure of the allosteric site in the α^Mg^γ^Mg+CIT+ADP^ structure. The key residues and the CIT and ATP (or ADP) are shown with ball-and-stick models, and the Mg^2+^ and water molecules are shown with green and red spheres, respectively. Hydrogen-bonding interactions of ATP (or ADP) and Mg^2+^ with CIT and the surrounding residues are indicated with dotted lines.

In both the α^Mg^γ^Mg+CIT+ATP^ and α^Mg+ATP^γ^Mg+CIT+ATP^ structures, there are Mg^2+^, CIT and ATP bound at the allosteric site, and the residues involved in the binding of the metal ion and ligands assume almost identical conformations as those seen in the α^Mg^γ^Mg+CIT+ADP^ structure **(**Fig. 2a**)**. Structural analysis shows that ATP occupies the ADP binding site, and particularly the adenine and α-phosphate of ATP make very similar interactions with the surrounding residues as those of ADP. In detail, the adenine of ATP forms two hydrogen bonds with the main-chain amine and carbonyl of Asn285^G^, and the α-phosphate forms two hydrogen bonds with the main-chain amines of Thr274^G^ and Gly275^G^ **(**Fig. 2b,c**)**. However, unlike the β-phosphate of ADP, which is oriented towards the Mg^2+^-binding site and forms two hydrogen bonds with the side chains of Asn273^G^ and Thr274^G^ and a coordination bond with the Mg^2+^ **(**Fig. 2d**)**, the β- and γ-phosphates of ATP are oriented away from the Mg^2+^-binding site and make different interactions with the enzyme due to potential steric conflicts with the surrounding residues. Specifically, the β-phosphate of ATP forms two hydrogen bonds with the main-chain amines of Lys276^G^ and Ser277^G^, and the γ-phosphate forms a hydrogen bond with the side chain of Ser277^G^ **(**Fig. 2b,c**)**. Meanwhile, a water molecule occupies the position of the β-phosphate of ADP and mediates the interaction between the Mg^2+^ and the α-phosphate of ATP. These results indicate that ATP can mimic ADP to bind to the allosteric site, and this ATP binding stabilizes the CIT and Mg^2+^ binding and thus the related conformational changes, leading the enzyme to adopt an active conformation. This provides the structural basis for why the αγ heterodimer can be activated by low concentrations of ATP. Since ATP has a larger volume than ADP, the ADP binding site cannot accommodate the three phosphates of ATP, and hence the β- and γ-phosphates of ATP are positioned away from the Mg^2+^-binding site. As a result, the interaction between ATP and Mg^2+^ is mediated via a water molecule instead of the direct interaction between ADP and Mg^2+^, suggesting that the stabilization of the CIT and Mg^2+^ binding by ATP is weaker than the stabilization by ADP. This explains why ATP has a weaker activatory effect than ADP^16,17^.

### ATP can also bind to the NAD binding site of the active site and thus inhibits the enzyme

**Figure 3:**
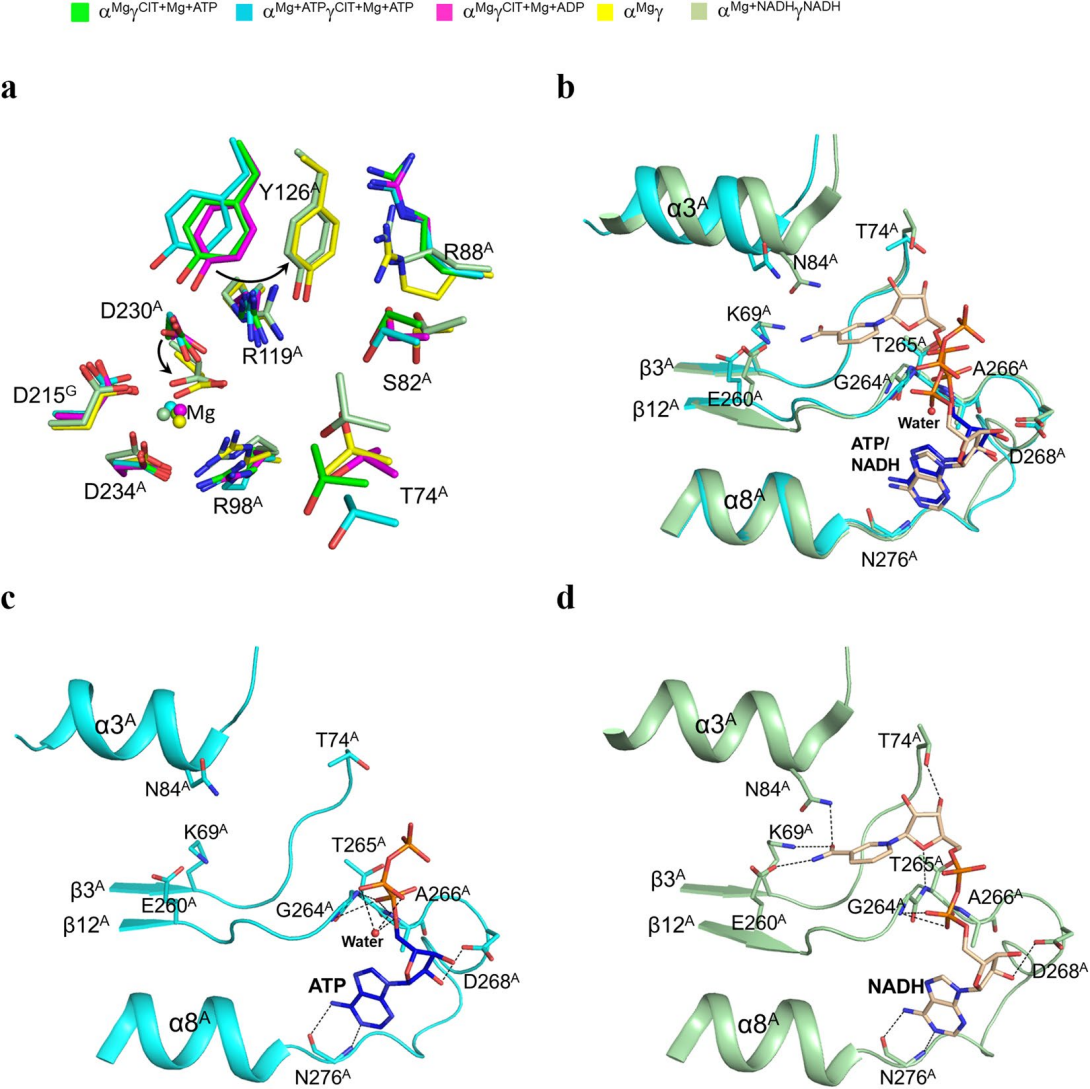
Structures of the active sites in the α^Mg^γ^Mg+CIT+ATP^ and α^Mg+ATP^γ^Mg+CIT+ATP^ structures. **(a)** Comparison of the active sites in the α^Mg^γ^Mg+CIT+ATP^, α^Mg+ATP^γ^Mg+CIT+ATP^, α^Mg^γ, α^Mg^γ^Mg+CIT+ADP^, and α^Mg+NADH^γ^NADH^ structures. The key residues of the active site in the α^Mg^γ^Mg+CIT+ATP^ and α^Mg+ATP^γ^Mg+CIT+ATP^ structures assume very similar conformations as those in the active α^Mg^γ^Mg+CIT+ADP^ structure rather than the inactive α^Mg^γ structure. The color scheme of the structures is shown above. **(b)** Comparison of the NAD binding sites in the α^Mg+ATP^γ^Mg+CIT+ATP^ and α^Mg+NADH^γ^NADH^ structures. The key residues of the NAD binding site in the α^Mg+ATP^γ^Mg+CIT+ATP^ structure assume very similar conformations as those in the α^Mg+NADH^γ^NADH^ structure. **(c)** Structure of the NAD binding site in the α^Mg+ATP^γ^Mg+CIT+ATP^ structure. **(d)** Structure of the NAD binding site in the α^Mg+NADH^γ^NADH^ structure. The key residues and the ATP (or NADH) are shown with ball-and-stick models, and the water molecule is shown with a red sphere. Hydrogen-bonding interactions of ATP (or NADH) with the surrounding residues are indicated with dotted lines.

In the α^Mg^γ^Mg+CIT+ATP^ structure, there is Mg^2+^ bound at the active site, and in the α^Mg+ATP^γ^Mg+CIT+ATP^ structure, there are both Mg^2+^ and ATP bound at the active site. Structural comparison shows that in both structures, the key residues at the active site assume very similar conformations, and the Mg^2+^ maintains identical interactions with the surrounding residues as those seen in the α^Mg^γ^Mg+CIT+ADP^ structure, rather than those seen in the α^Mg^γ structure **(**Fig. 3a**)**. As there is no structure of the αγ heterodimer bound with an NAD at the active site thus far, we compared the α^Mg+ATP^γ^Mg+CIT+ATP^ structure with the α^Mg+NADH^γ^NADH^ structure to dissect the effects of the ATP binding at the active site. The structural comparison shows that ATP binds to the NAD binding site, and the adenine, ribose, and α-phosphate of ATP occupy the positions of the corresponding moieties of NADH in the α^Mg+NADH^γ^NADH^ structure and maintain similar interactions with the surrounding residues^19^ **(**Fig. 3b,c**)**. In detail, the adenine of ATP forms two hydrogen bonds with the main-chain amine and carbonyl of Asn276^A^, the ribose 2′-OH forms a hydrogen bond with the side chain of Asp268^A^, and the α-phosphate forms two hydrogen bonds with the main-chain amines of Gly264^A^ and Thr265^A^. On the other hand, the β- and γ-phosphates of ATP occupy a slightly different position from the nicotinamide moiety of NADH and make no interactions with any residues of the enzyme; however, there is a water molecule mediating the interaction between the α- and β-phosphates of ATP. The observation that ATP makes fewer interactions with the enzyme compared to NADH (and NAD) suggests that ATP has a weaker binding with the enzyme than NADH (and NAD). These results indicate that ATP at high concentrations can mimic NADH (and NAD) to bind to the NAD binding site and prevents NAD binding which accounts for in part why high concentrations of ATP can inhibit the enzymatic activity of the αγ heterodimer.

### ATP facilitates the formation of the MnATP or MgATP chelate and decreases the concentration of free metal ion available for the catalytic reaction

**Figure 4:**
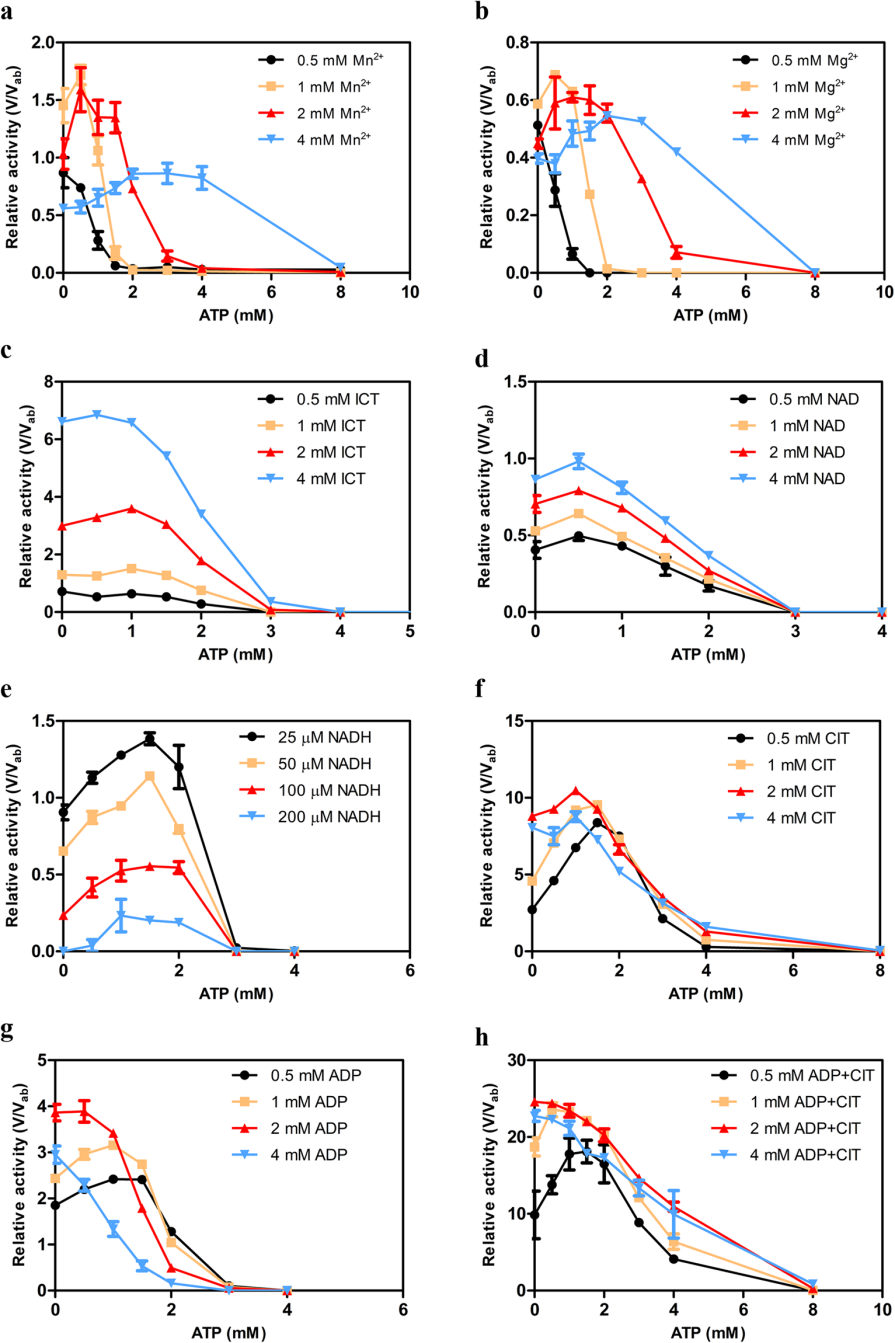
Effects of the metal ion and different ligands on the dual regulatory roles of ATP on the αγ heterodimer. **(a)** Effect of Mn^2+^ on the dual regulatory roles of ATP. **(b)** Effect of Mg^2+^ on the dual regulatory roles of ATP. **(c)** Effect of the substrate ICT on the dual regulatory roles of ATP. **(d)** Effect of the cofactor NAD on the dual regulatory roles of ATP. **(e)** Effect of the inhibitor NADH on the dual regulatory roles of ATP. **(f)** Effect of the activator CIT on the dual regulatory roles of ATP. **(g)** Effect of the activator ADP on the dual regulatory roles of ATP. **(h)** Effect of the activators CIT and ADP on the dual regulatory roles of ATP. The activities were measured at the standard conditions with varied concentrations of ATP in the absence and presence of different concentrations of the metal ion or ligand(s). The regulatory effect of ATP is presented as the ratio (V/V_ab_) of the enzymatic activity in the presence of ATP (and regulators) (V) *verse* that in the absence of ATP and any regulators at the standard conditions (V_ab_ which is defined as 1). The values are the averages of two independent measurements with the standard errors.

In solution, ATP and ADP form chelates with many metal cations (in particular Mg^2+^)^21–23^. In cells, about 90% of total intracellular ATP (in the range of 1–10 mM depending on the cell types and determination methods) exists in the form of MgATP, and about 50% of total intracellular ADP (in the range of 0.1–1 mM) exists in the form of MgADP^24–27^. Thus, the concentrations of ATP and ADP can affect the proportion of the metal ion that exists in chelate form versus free ion form, thereby modulating the enzymatic activity in many metal dependent catalytic reactions. We speculated that high concentrations of ATP may promote the formation of MnATP or MgATP and decrease the concentration of free Mn^2+^ or Mg^2+^ available for the catalytic reaction, leading to the inhibition of the enzyme. To investigate this possibility, we analyzed the dual regulatory roles of ATP on the activity of the αγ heterodimer at different concentrations of Mn^2+^ or Mg^2+^ (0.5 mM, 1 mM, 2 mM, and 4 mM) and in the absence of the activators CIT and ADP (Fig. 4a,b). The biochemical data show that the enzyme exhibits a higher activity (roughly 2 fold higher) in the presence of Mn^2+^ compared to Mg^2+^, consistent with our previous biochemical data showing that Mn^2+^ is the most effective metal ion for the αγ heterodimer and the holoenzyme, followed by Mg^2+16^. At 0.5 mM Mn^2+^ or Mg^2+^, ATP has only inhibitory effect. However, at higher concentrations of Mn^2+^ or Mg^2+^ (1 mM, 2 mM, and 4 mM), ATP has activatory effect at low concentrations but inhibitory effect at high concentrations. Moreover, as the metal ion concentration increases, the ATP concentrations required to reach the optimal activation and the complete inhibition of the enzyme are elevated; additionally, the ATP concentration range for activation is also extended (Fig. 4a,b). These results indicate that high concentrations of ATP facilitate the formation of metal ion-ATP chelate and thus inhibit the enzymatic activity. Conversely, the increase in the metal ion concentration increases the availability of free metal ion for the catalytic reaction, and thus compensates for the inhibitory effect of high ATP concentrations.

To investigate whether ATP may exert the dual regulatory roles through interplay(s) with other ligand(s), we also analyzed the regulatory effect of ATP on the αγ heterodimer at different concentrations of the substrate ICT, the cofactor NAD, the inhibitor NADH, and the activators CIT and ADP (Fig. 4c–h). As expected, as the concentration of ICT or NAD increases, the enzymatic activity is increased accordingly; however, the regulatory pattern of ATP does not change, and the ATP concentrations required to reach the optimal activation and the complete inhibition of the enzyme do not change either. This indicates that the dual regulatory roles of ATP are not affected by the substrate and the cofactor (Fig. 4c,d). As the concentration of NADH increases, the enzymatic activity is gradually decreased, but the regulatory pattern of ATP does not change. This indicates that NADH has only inhibitory effect on the enzyme, and the concentration of NADH has no notable effect on the dual regulatory roles of ATP (Fig. 4e).

On the other hand, in the presence of varying concentrations of CIT, ATP always plays dual regulatory roles, and the ATP concentration required to reach the optimal activation is slightly lowered from 1.5 mM ATP in the presence of lower concentrations of CIT (0.5 mM and 1 mM) to 1 mM ATP in the presence of higher concentrations of CIT (2 mM and 4 mM) (Fig. 4f). Intriguingly, unlike what we found when varying ICT and NAD, the enzymatic activity is gradually increased at lower concentrations of CIT (0.5 mM, 1 mM and 2 mM) but is decreased at a higher concentration of CIT (4 mM). These results are consistent with our previous biochemical data showing that at the standard conditions, the concentration of CIT necessary to reach the optimal activation is about 2 mM^19^. This might be explained as follows: when the CIT concentration reaches the optimal activation level (2 mM), more CIT would compete with ICT to bind to the active site, and thus inhibit the enzymatic activity. In the presence of lower concentrations of ADP (0.5 mM, 1 mM and 2 mM), ATP plays dual regulatory roles, and the ATP concentration required to reach the optimal activation is gradually decreased from 1.5 mM ATP (at 0.5 mM ADP) to 0.5 mM (at 2 mM ADP); meanwhile, the enzymatic activity is also increased accordingly (Fig. 4g). In the presence of a higher concentration of ADP (4 mM), ATP has only inhibitory effect, and the activity is decreased as well. These results are also consistent with our previous biochemical data showing that at the standard conditions, the concentration of ADP necessary to reach the optimal activation is about 2 mM^19^. This could be explained as follows: ATP can mimic ADP to activate the enzyme, and when the total concentration of ADP and ATP reaches the optimal activation level (about 2 mM), a further increase in ADP and ATP would compete with NAD to bind to the active site or reduce the concentration of free metal ion, thus inhibiting the enzymatic activity. As expected, in the presence of both CIT and ADP, ATP exhibits a regulatory pattern similar to a combination of the patterns seen in the presence of CIT and ADP alone (Fig. 4h). In the presence of lower concentrations of CIT and ADP (0.5 mM and 1 mM), ATP plays dual regulatory roles; the ATP concentration required to reach the optimal activation is gradually decreased from about 1.5 mM ATP (at 0.5 mM ADP) to 1 mM (at 1 mM ADP), and the enzymatic activity is gradually increased. In the presence of higher concentrations of CIT and ADP (2 mM and 4 mM), ATP has only inhibitory effect, and the enzymatic activity is gradually decreased. Taken together, these results indicate that low concentrations of ATP have a synergistic activatory effect with CIT; however, this activatory effect is compromised by ADP because ADP has a tighter binding to the allosteric site compared to ATP.

## Discussion

In cells, the ATP/ADP ratio is considered to be a key factor for controlling respiration and metabolism in response to energy demand. A low ATP/ADP ratio corresponds to a low energy level of the cell and thus attenuates cellular respiration, whereas a high ATP/ADP ratio corresponds to a high energy level of the cell and thus ramps down cellular respiration. As a rate-limiting step of the Krebs cycle, the activity of NAD-IDH is also regulated by ADP and ATP. Our previous biochemical and structural studies and the early biochemical studies of other groups showed that ADP can positively regulate the activity of mammalian NAD-IDHs via an allosteric mechanism^10,12–17^. Intriguingly, while the early biochemical studies showed that ATP has inhibitory effect on the activity of mammalian NAD-IDHs^15,20^, our biochemical studies showed that ATP can activate the activities of the αγ heterodimer and the holoenzyme of human NAD-IDH at low concentrations but inhibit the enzymatic activity at high concentrations. To investigate the molecular basis for the dual regulatory roles of ATP on the αγ heterodimer, we determined the crystal structure of α^Mg^γ^Mg+CIT+ATP^, which was obtained from crystals grown from a crystallization solution containing a low concentration of ATP (2 mM), and we also determined the crystal structure of α^Mg+ATP^γ^Mg+CIT+ATP^, which was obtained from soaking the crystals of α^Mg^γ^Mg+CIT^ in a crystallization solution containing a high concentration of ATP (200 mM). In addition, we performed biochemical studies to analyze the effects of the concentrations of metal ion and ligands on the dual regulatory roles of ATP on the αγ heterodimer. The structural and biochemical data together reveal the molecular mechanisms of the activation and inhibition of the αγ heterodimer by ATP.

In both the α^Mg^γ^Mg+CIT+ATP^ and α^Mg+ATP^γ^Mg+CIT+ATP^ structures, there are Mg^2+^, CIT and ATP bound to the allosteric site. The ATP mimics ADP in binding to the ADP binding site; this ATP binding stabilizes the CIT and Mg^2+^ binding and the associated conformational changes at the allosteric site, the active site, and the heterodimer interface, leading the enzyme to adopt an active conformation similar to that of α^Mg^γ^Mg+CIT+ADP^. As the ADP binding site cannot accommodate the three phosphates of ATP, the β- and γ-phosphates of ATP are oriented away from the Mg^2+^-binding site, and the stabilization of the CIT and Mg^2+^ binding by ATP is mediated via a water molecule, which is apparently weaker than the stabilization by ADP. The structural results are consistent with our previous and current biochemical data showing that both ADP and ATP have a synergistic activatory effect with CIT on the αγ heterodimer; however, the activatory effect of ATP is weaker than that of ADP, and thus can be compromised by ADP. The structural and biochemical data together provide the molecular basis to explain why the αγ heterodimer can be activated by low concentrations of ATP.

On the other hand, the inhibition of the αγ heterodimer by high concentrations of ATP occurs through two different mechanisms. In the α^Mg+ATP^γ^Mg+CIT+ATP^ structure, there are Mg^2+^ and ATP bound to the active site. The ATP mimics NADH in binding to the NAD binding site; this binding prevents the binding of NAD and leads to the inhibition of the enzyme. Nevertheless, although the adenine, the ribose, and the α-phosphate of ATP occupy the same positions as the corresponding moieties of NADH in the α^Mg+NADH^γ^NADH^ structure and maintain similar interactions with the surrounding residues^19^, the β- and γ-phosphates of ATP occupy a slightly different position from the nicotinamide moiety of NADH and make no interactions with the enzyme. This suggests that ATP has a weaker binding with the enzyme compared to NADH (and NAD). These results are also consistent with the structural data demonstrating that ATP can only bind to the NAD binding site at a very high concentration. Moreover, our biochemical data show that increases in the metal ion concentration can increase the ATP concentration required to reach the optimal activation and the complete inhibition of the enzyme, as well as broaden the ATP concentration range for activation. This indicates that increasing the metal ion concentration can compensate for the inhibitory effect of ATP. These results suggest that high concentrations of ATP facilitate the formation of MgATP or MnATP and reduce the concentration of free metal ion available for the catalytic reaction, leading to the inhibition of the enzyme. The combined effects of the competitive binding of ATP to the NAD binding site and the formation of ATP-metal chelate account for the inhibition of the αγ heterodimer by high concentrations of ATP.

## Materials and methods

### Cloning, expression and purification

The αγ heterodimer of human NAD-IDH was prepared as described previously^16^. Briefly, the DNA fragments encoding the α and γ subunits of human NAD-IDH were cloned into co-expression vector pQlinkN with the C-terminal of the γ subunit attached with a TEV protease cleavage site and a His_6_ tag following the pQlinkN cloning procedure^28^. The pQlinkN-α-γ-tev-His_6_ plasmid was transformed into *E. coli* BL21(DE3) CodonPlus strain (Novagen), and protein expression was induced by 0.4 mM IPTG for 20 hours at 23 °C. The target protein was first purified by affinity chromatography using an Ni-NTA column (Qiagen). The His_6_-tag of the target protein was cleaved by TEV protease, and the protein mixture was reloaded on an Ni-NTA column and the flow-through fraction containing the target protein was further purified by gel filtration using a Superdex 200 10/300 GL column (GE Healthcare). The purified protein has high purity (>95%) as analyzed by 12% SDS-PAGE, which was concentrated to about 10 mg/ml and stored in the storage buffer (10 mM HEPES, pH 7.4, 200 mM NaCl, and 5 mM β-ME) for the structural and biochemical studies.

### Crystallization, diffraction data collection, structure determination and refinement

Crystallization was performed using the hanging drop vapor diffusion method at 20 °C. Crystals of the α^Mg^γ^Mg+CIT+ATP^ heterodimer were grown from drops consisting of equal volumes (1 μl) of the protein solution supplemented with 0.2 mM Mg^2+^ and 2 mM ATP, and the reservoir solution containing 0.2 M sodium citrate, pH 8.0, and 20% (w/v) PEG3350. Crystals of the α^Mg^γ^Mg+CIT^ heterodimer were grown from drops consisting of the protein solution supplemented with 0.2 mM Mg^2+^, and the reservoir solution containing 0.2 M sodium citrate, pH 5.5, and 24% (v/v) PEG400. Crystals of the α^Mg+ATP^γ^Mg+CIT+ATP^ heterodimer were obtained by soaking the crystals of α^Mg^γ^Mg+CIT^ in the crystallization solution supplemented with 200 mM ATP for 10 mins. The pH values of the stock solutions of the metal ion and ATP were adjusted to 7.4, which is the same as that of the protein stock solution, before addition into the crystallization or soaking solutions. Prior to diffraction data collection, the crystals were cryoprotected using the reservoir solution supplemented with 25% ethylene glycol and then flash-cooled into liquid N_2_. Diffraction data were collected at 100 K at BL17U1 of Shanghai Synchrotron Radiation Facility (SSRF) and BL19U1 of National Facility for Protein Science in Shanghai (NFPSS) and processed with HKL2000^29^. Statistics of the diffraction data are summarized in Table 1.

The α^Mg^γ^Mg+CIT+ATP^ and α^Mg+ATP^γ^Mg+CIT+ATP^ structures were solved with the molecular replacement method as implemented in program Phaser^30^ using the α^Mg^γ structure (PDB code 5GRH) as the search model. Structure refinement was carried out initially with program Phenix^31^ and finally with program REFMAC5^32^. Manual model building was carried out with program Coot^33^. Stereochemistry and quality of the structure models were analyzed using programs in the CCP4 suite^34^. Structure figures were prepared using PyMol^35^. Statistics of the structure refinement and final structure models are summarized in Table 1.

### Enzymatic activity assay and kinetic analysis

Enzymatic activity of the αγ heterodimer was assayed using a method as described previously^16^. The standard reaction solution (1 ml) consisted of 33 mM Tris-acetate, pH 7.4, 2 μg enzyme, 0.6 mM ICT, 2 mM Mn^2+^, and 3.2 mM NAD. The activity is defined as the velocity (V) at the specific conditions, which is expressed as μmol of NADH produced per min per mg of enzyme (μmol/min/mg). To analyze the dual regulatory roles of ATP on the activity, the activity was measured at the standard conditions with varied concentrations of ATP (0–10 mM). The pH value of the ATP stock solution was adjusted to 7.4, which is the same as that of the protein stock solution, before addition into the reaction mixture. The effect of ATP is presented as the ratio (V/V_ab_) of the activity of the enzyme in the presence of ATP (and regulators) (V) *verse* that in the absence of ATP and any regulators at the standard conditions (V_ab_ which is defined as 1).

To analyze the effects of Mn^2+^ and Mg^2+^ on the dual regulatory roles of ATP, the activity was measured at the standard conditions with varied concentrations of Mn^2+^ or Mg^2+^ (0.5 mM, 1 mM, 2 mM, and 4 mM). To analyze the effects of the substrate ICT and the cofactor NAD on the dual regulatory roles of ATP, the activity was measured at the standard conditions with varied concentrations of ICT (0.5 mM, 1 mM, 2 mM, and 4 mM) or NAD (0.5 mM, 1 mM, 2 mM, and 4 mM). To analyze the effect of the inhibitor NADH on the dual regulatory roles of ATP, the activity was measured at the standard conditions with varied concentrations of NADH (25 μM, 50 μM, 100 μM, and 200 μM). To analyze the effects of the activators CIT or/and ADP on the dual regulatory roles of ATP, the activity was measured at the standard conditions with varied concentrations of CIT (0.5 mM, 1 mM, 2 mM, and 4 mM), or ADP (0.5 mM, 1 mM, 2 mM, and 4 mM), or both CIT and ADP. The pH values of the stock solutions of the metal ion and ligands were adjusted to 7.4, which is the same as that of the protein stock solution, before addition into the reaction mixture. All experiments were carried out at 25 °C and repeated at least twice under the same conditions.

### Protein data bank accession codes

The α^Mg^γ^Mg+CIT+ATP^ and α^Mg+ATP^γ^Mg+CIT+ATP^ structures of human NAD-IDH have been deposited in the Protein Data Bank with accession codes 6L57 and 6L59, respectively.

## References

[1] Jo SH (2001). Control of mitochondrial redox balance and cellular defense against oxidative damage by mitochondrial NADP^+^-dependent isocitrate dehydrogenase. J. Biol. Chem..

[2] Lee SM (2002). Cytosolic NADP^+^-dependent isocitrate dehydrogenase status modulates oxidative damage to cells. Free Radic. Biol. Med..

[3] Kim SY, Park JW (2003). Cellular defense against singlet oxygen-induced oxidative damage by cytosolic NADP^+^-dependent isocitrate dehydrogenase. Free Radic. Res..

[4] Koh HJ (2004). Cytosolic NADP^+^-dependent isocitrate dehydrogenase plays a key role in lipid metabolism. J. Biol. Chem..

[5] Xu X (2004). Structures of human cytosolic NADP-dependent isocitrate dehydrogenase reveal a novel self-regulatory mechanism of activity. J. Biol. Chem..

[6] Ceccarelli C, Grodsky NB, Ariyaratne N, Colman RF, Bahnson BJ (2002). Crystal structure of porcine mitochondrial NADP^+^-dependent isocitrate dehydrogenase complexed with Mn^2+^ and isocitrate: insights into the enzyme mechanism. J. Biol. Chem..

[7] Yang B, Zhong C, Peng Y, Lai Z, Ding J (2010). Molecular mechanisms of “off-on switch” of activities of human IDH1 by tumor-associated mutation R132H. Cell Res..

[8] Nichols BJ, Hall L, Perry AC, Denton RM (1993). Molecular cloning and deduced amino acid sequences of the γ-subunits of rat and monkey NAD^+^-isocitrate dehydrogenases. Biochem. J..

[9] Nichols BJ, Perry AC, Hall L, Denton RM (1995). Molecular cloning and deduced amino acid sequences of the α- and β- subunits of mammalian NAD^+^-isocitrate dehydrogenase. Biochem. J..

[10] Ehrlich RS, Colman RF (1981). Binding of ligands to half of subunits of NAD-dependent isocitrate dehydrogenase from pig heart. Binding of manganous ion, isocitrate, ADP and NAD. J. Biol. Chem..

[11] Ehrlich RS, Colman R (1983). Separation, recombination, and characterization of dissimilar subunits of the DPN-dependent isocitrate dehydrogenase from pig heart. J. Biol. Chem..

[12] Cohen PF, Colman RF (1972). Diphosphopyridine nucleotide dependent isocitrate dehydrogenase from pig heart. Charactgerization of the active substrate and modes of regulation. Biochemistry.

[13] Gabriel JL, Plaut GW (1984). Inhibition of bovine heart NAD-specific isocitrate dehydrogenase by reduced pyridine nucleotides: modulation of inhibition by ADP, NAD^+^, Ca^2+^, citrate, and isocitrate. Biochemistry.

[14] Gabriel J, Plaut G (1984). Citrate activation of NAD-specific isocitrate dehydrogenase from bovine heart. J. Biol. Chem..

[15] Gabriel JL, Milner R, Plaut GW (1985). Inhibition and activation of bovine heart NAD-specific isocitrate dehydrogenase by ATP. Arch. Biochem. Biophys..

[16] Ma T, Peng Y, Huang W, Liu Y, Ding J (2017). The β and γ subunits play distinct functional roles in the *α*_2_βγ heterotetramer of human NAD-dependent isocitrate dehydrogenase. Sci. Rep..

[17] Ma T, Peng Y, Huang W, Ding J (2017). Molecular mechanism of the allosteric regulation of the *α*γ heterodimer of human NAD-dependent isocitrate dehydrogenase. Sci. Rep..

[18] Sun P (2019). Molecular basis for the function of the *α*β heterodimer of human NAD-dependent isocitrate dehydrogenase. J. Biol. Chem..

[19] Liu Y, Hu L, Ma T, Yang J, Ding J (2018). Insights into the inhibitory mechanisms of NADH on the *α*γ heterodimer of human NAD-dependent isocitrate dehydrogenase. Sci. Rep..

[20] Chen RF, Plaut G (1963). Activation and inhibition of DPN-linked isocitrate dehydrogenase of heart by certain nucleotides. Biochemistry.

[21] Osullivan WJ, Perrin DD (1964). Stability constants of metal-adenine nucleotide complexes. Biochemistry.

[22] Jahngen JH, Rossomando EF (1983). Resolution of an ATP-metal chelate from metal-free ATP by reverse-phase high-performance liquid-chromatography. Anal. Biochem..

[23] Wilson JE, Chin A (1991). Chelation of divalent-cations by ATP, studied by titration calorimetry. Anal. Biochem..

[24] Gout E, Rebeille F, Douce R, Bligny R (2014). Interplay of Mg^2+^, ADP, and ATP in the cytosol and mitochondria: unravelling the role of Mg^2+^ in cell respiration. Proc. Natl. Acad. Sci. USA.

[25] Li HY, Dai LJ, Krieger C, Quamme GA (1993). Intracellular Mg^2+^ concentrations following metabolic inhibition in opossum kidney cells. Biochim. Biophys. Acta..

[26] Fang BY (2016). Detection of adenosine triphosphate in HeLa cell using capillary electrophoresis-laser induced fluorescence detection based on aptamer and graphene oxide. Colloids. Surf. B Biointerfaces.

[27] Gupta RK, Yushok WD (1980). Noninvasive 31P NMR probes of free Mg^2+^, MgATP, and MgADP in intact Ehrlich ascites tumor cells. Proc. Natl. Acad. Sci. USA.

[28] Scheich C, Kümmel D, Soumailakakis D, Heinemann U, Büssow K (2007). Vectors for co-expression of an unrestricted number of proteins. Nucleic acids Res..

[29] Otwinowski Z (1997). M. W. Processing of X-ray diffraction data collected in oscillation mode. Methods Enzymol..

[30] McCoy AJ (2007). Phaser crystallographic software. J. Appl. Crystallogr..

[31] Adams PD (2010). PHENIX: a comprehensive Python-based system for macromolecular structure solution. Acta Crystallogr. D Biol.Crystallogr..

[32] Murshudov GN, Vagin AA, Dodson EJ (1997). Refinement of macromolecular structures by the maximum-likelihood method. Acta Crystallogr. D Biol. Crystallogr..

[33] Emsley P, Cowtan K (2004). Coot: model-building tools for molecular graphics. Acta Crystallogr. D Biol. Crystallogr..

[34] Winn MD (2011). Overview of the CCP4 suite and current developments. Acta Crystallogr. D Biol. Crystallogr..

[35] Schrodinger, LLC. *The PyMOL Molecular Graphics System*, *Version* 1.*3r1* (2010).

